# Disruption of the *ECM33* Gene in *Candida albicans* Prevents Biofilm Formation, Engineered Human Oral Mucosa Tissue Damage and Gingival Cell Necrosis/Apoptosis

**DOI:** 10.1155/2012/398207

**Published:** 2012-05-14

**Authors:** Mahmoud Rouabhia, Abdelhabib Semlali, Jyotsna Chandra, Pranab Mukherjee, Witold Chmielewski, Mahmoud A. Ghannoum

**Affiliations:** ^1^Groupe de Recherche en Écologie Buccale, Faculté de Médecine Dentaire, Université Laval Pavillon de Médecine Dentaire, 2420 Rue de la Terrasse, QC, Canada G1V 0A6; ^2^Center for Medical Mycology, Department of Dermatology, School of Medicine, Case Western Reserve University and University Hospitals Case Medical Center, Cleveland, OH 44106, USA

## Abstract

In this study we demonstrated that Δ*Caecm33* double mutant showed reduced biofilm formation and causes less damage to gingival mucosa tissues. This was confirmed by the reduced level of necrotic cells and Bax/Bcl2 gene expression as apoptotic markers. In contrast, parental and *Caecm33* mutant strains decreased basement membrane protein production (laminin 5 and type IV collagen). We thus propose that *ECM33* gene/protein represents a novel target for the prevention and treatment of infections caused by *Candida*.

## 1. Introduction

*Candida* species are the most frequent cause of life-threatening invasive fungal infections in the immunocompromised host [1, 2]. Under predisposing conditions, *C. albicans* multiplies and penetrates the host tissue to cause inflammation and tissue destruction [3]. *C. albicans* adherence to host cells is therefore the first step in the initiation of infection, as this enables the organism to evade the normal flushing mechanisms of body secretions [4].

The* Candida* cell wall is the initial point of contact and interaction with host tissues. This wall consists of a complex structure that houses a network of polysaccharides (primarily *β*-glucans and chitin) in which various proteins such as glycosylphosphatidylinositol (GPI) interact [5]. The key function of GPI proteins in *Candida* biogenesis and maintenance of the fungus cell wall involves *ECM33 *gene [6]. ECM33 protein is required for normal cell wall integrity and the yeast-to-hyphae transition *in vitro* [7]. *C. albicans Caecm33*Δ*/Caecm33*Δ mutant showed blastospores that were flocculated and were larger than those of the wild-type strain. *Caecm33*Δ*/Caecm33*Δ mutant displayed delayed hyphal formation and showed attenuated virulence in the mouse model of hematogenously disseminated candidiasis [8].

The absence of *ECM33* gene may also prevent *Candida* from affecting the oral mucosa. Oral epithelium interacts with connective tissue via basement membrane (BM) proteins that contribute to body integrity.

Oral mucosa contains a highly complex stratified epithelia that protects the body from physical and chemical damage, infection, dehydration, and heat loss through interactions with the mesenchymal tissue via basement membrane (BM) proteins [9–11]. The BM is a thin layer of complex extracellular matrix that forms the support structure on which epithelial cells grow. This layer also provides mechanical support, divides tissue into compartments, and significantly influences cellular behavior [12]. Two major components of the BM are type IV collagen and laminin. Type IV collagen forms a network that confers the distinctive mechanical stability known to the BM [13]. Laminin binds to collagen IV to constitute a second network by interacting with nidogen. Laminin 5 is specific to the basement membrane underlying the squamous epithelium and mucosa [14]. Its primary role is to modulate stable epithelial cell attachment through interactions with integrins *α*_3_ *β*_1_ and *α*_6_ *β*_4_ [15].

The interaction between *Candida* and the oral epithelium is believed to be one of the most important initial events in the prevention or development of candidiasis [18]. However, following contact with *C. albicans*, and under certain circumstances such as reduced innate immunity, *C. albicans* may occasionally become pathogenic and induce lesions on the oral mucosa [19, 20]. This may occur through a deregulation of BM protein synthesis and deposition, which in turn induces a breakdown of oral homeostasis and, consequently, systemic infection [21, 22]. Tissue structure and BM protein deregulation may occur through apoptotic processes involving inducer (Bax, Bcl-xS, Bad) and inhibitor (Bcl-2, Bcl-xL) genes [23]. Indeed, host cell death following microbial infection is typically recognized as necrotic, apoptotic, or pyroptotic [24–26]. While necrosis is characterized as accidental cell death as the result of physical damage, apoptosis and pyroptosis are strictly regulated genetic and biochemical self-destruction programs that are critical during development and tissue homeostasis as well as in modulating the pathogenesis of a variety of infectious diseases [27]. A recent study reported that *C. albicans* stimulated the oral epithelial signaling pathways that promote early apoptotic cell death through the activation of cellular caspases, followed by late necrosis [22].

Considering the key role of oral mucosa in preventing/controlling *Candida* pathogenesis, and given the adhesion, which is the first stage of biofilm formation of *Candida* to the tissue through specific proteins such as *ECM33*, we sought to investigate the role of *ECM33* gene on *Candida* biofilm formation, and its interaction with oral mucosa tissue. The central hypothesis of this study is that *ECM33* gene is involved in *Candida* virulence leading to tissue damage and facilitating the onset of candidiasis. To test this hypothesis, we examined the ability of *ECM*33 isogenic strains of *C. albicans* to form biofilms *in vitro *on a catheter-associated biofilm model in order to induce tissue damage, cell necrosis, apoptosis signalling molecule (Bax and Bcl2) activation, and laminin 5 and type IV collagen modulation. To move our study closer to the clinical setting, we used an engineered human oral mucosa model, as previously described [4].

## 2. Materials and Methods

### 2.1. Candida Strains

Table 1 presents the *Candida albicans* strains used in this study. These strains were generously donated by Dr. C. Gil (Madrid, Spain). The parental (CAF2) *C. albicans* strain was genotypically identified as *URA3*/Δ*ura3*::*imm43* [16]. Each *Candida* strain was cultured on Sabouraud dextrose agar plates (Becton Dickinson, Oakville, ON, Canada) at 30°C. To prepare the *C. albicans* suspension, one colony was used to inoculate 10 mL of Phytone-peptone medium (Becton Dickinson) supplemented with 0.1% glucose and 80 mg/L of uridine at pH 5.6. The cultures were grown under shaking conditions in a water bath for 18 h, after which time the *Candida* cells were collected, washed with PBS, and resuspended in the same buffer to a density of 1 × 10^7^ cells/mL to obtain a standardized cell suspension.

### 2.2. Catheter-Associated *C. albicans* Biofilm Formation

The first step was to prepare the substrate material (silicone elastomer) for biofilm formation. Silicone elastomer (SE) sheets were obtained from Invotec International (Jacksonville, FL, USA). Following the manufacturer's instructions, the catheter material sheet was cleaned by scrubbing it thoroughly with a clean, soft-bristled brush in a hot water/hand soap solution, rinsed with distilled water, and autoclaved. Flat circular discs 15 mm in diameter were obtained by cutting through the catheter sheets with a cork borer, as previously described [28]. The sterile SE disks were distributed into 12-well plates, precoated with fetal bovine serum (Mediatech, VA, USA) for 24 h at 37°C on a rocker table and then exposed to one of the *Candida* strains (1 × 10^7^ cells/mL). To assist *C. albicans* adhesion, the SE discs were incubated at 37°C for 90 min, transferred to a sterile 12-well plate containing 4 mL of yeast nitrogen base medium, and subsequently cultured at 37°C for 48 h. Biofilm formation was quantified by means of a tetrazolium XTT [2,3-bis(2-methoxy-4-nitro-5-sulfophenyl)-2H-tetrazolium-5-carboxanilide] reduction assay [28]. Discs containing no *Candida* cells served as controls. To confirm biofilm formation, the *Candida*-seeded SE discs were transferred to microscope slides, stained with calcofluor-white (0.05% (vol/vol)) and examined under a fluorescence microscope at *λ*_max⁡_ = 432 nm.

### 2.3. Engineering Human Oral Mucosa Production

Gingival mucosa samples were biopsied from healthy donors following their informed consent and approval by the University Lavals ethics committee. Gingival fibroblasts and epithelial cells were subsequently extracted [29]. Following culture, the cells were used to engineer human oral mucosa (EHOM). The fibroblasts were mixed with bovine skin collagen to produce the lamina propria. Four days later, the epithelial cells were seeded on the lamina propria and grown until confluence. The EHOM was then raised to the air-liquid interface for five days for epithelium stratification and was used thereafter to study *C. albicans* pathogenesis.

### 2.4. Tissue Structure following Infection

EHOM was first grown in a serum-free, antifungal-free DMEH medium for 48 h and then placed in contact with one of the *C. albicans* (10^4^ cells/cm^2^) strains for 24 h. Noninfected EHOM was used as the control (Ctrl). Following infection and culture, the biopsies were collected, fixed with 4% paraformaldehyde solution, embedded in paraffin, and stained with hematoxylin-eosin for analysis.

### 2.5. Quantitation of the Biofilms Formed on the EHOM Tissue

To quantitatively assess the differences in biofilm formation by the selected *C. albicans* strains, multiple specimens were collected from each infected EHOM and stained with hematoxylin-eosin. Each slide was assigned a number, and measurements were made independently by two different observers. Each blind observer measured the thickness of *C. albicans* biofilms at regular intervals using a calibrated image analysis system (Image-Pro Plus software, Media Cybernetics, MD, USA), as we previously reported [30]. Ten measurements were performed on the unfolded area of each slide. We then compared the thickness of the biofilms formed on the EHOM specimens infected with the mutant strains and that of the biofilms formed by the wild-type and revertant strains. A *P* value of <0.05 was considered statistically significant (*n* = 4).

### 2.6. Lactate Dehydrogenase Assay

The release of lactate dehydrogenase (LDH) from the EHOM into the surrounding medium was monitored as a measure of tissue damage/cell necrosis. LDH activity release into the medium of the infected tissue (IT) was measured at 24 h using a Cytox96 kit (Promega, Madison, WI, USA) according to the manufacturer's instructions. A positive control (PC) was obtained by culturing the cells for one hour in the presence of 1% Triton X-100, while a negative control (NC) was obtained by culturing cells not exposed to any *Candida* strain. LDH activity was calculated as the percetage of total LDH activity release = (IT_absorbance_ − NC_absorbance_)/(PC_absorbance_ − NC_absorbance_) × 100. Differences between groups were determined by an ANOVA Student *t*-test and was considered significant at a *P* value of <0.05 (*n* = 4).

### 2.7. Apoptotic (Bax) and Antiapoptotic (Bcl-2) Gene Expression and Protein Production

Following EHOM infection with the *Candida* strains, total RNA was extracted from each EHOM and used to comparatively analyze Bax and Bcl2 gene expression by qRT-PCR [31] and GAPDH gene expression.  Table 2 presents the specific primer sequences used in this study. The thermocycling conditions for the Bax gene were 95°C for 5 min, followed by 40 cycles of 95°C for 15 s, 64°C for 30 s, and 72°C for 30 s, while those for the Bcl2 gene were 95°C for 3 min, followed by 40 cycles of 95°C for 10 s, 64°C for 10 s, and 72°C for 30 s. The specificity of each primer pair was verified by the presence of a single melting temperature peak. GAPDH produced uniform expression levels varying by less than 0.5 CTs between sample conditions and was therefore used as a reference gene for this study. Gene expression was supported by Bax and Bcl2 protein analyses. *Candida*-infected EHOM was used to prepare protein cell lysates which were then subjected to SDS polyacrylamide gel electrophoresis and electroblotting onto PVDF membranes [31]. The membranes were then blocked with 5% BSA in Tween-20/Tris-buffered saline (TTBS) for 1 h and were incubated overnight at 4°C with either primary anti-Bax or anti-Bcl2 antibodies (1 : 1000). After washing, the membranes were incubated with horseradish peroxidase-labeled secondary antibody (1 : 1000) for 1 h. For protein detection, the membranes were washed for 3 h with TBS, incubated in ECL, and analyzed on a Fujifilm Image Reader LAS-1000 Pro.

### 2.8. Laminin 5 and Type IV Collagen Protein Production

Following contact for 24 h with one of the *Candida* strains, the EHOM protein lysates were used to assess laminin 5 and type IV collagen production by western blotting using mouse antilaminin 5 (1 : 200) or mouse antitype IV collagen (1 : 1000). Each membrane was washed in TBS-T then incubated with the appropriate secondary horseradish peroxidase-labeled antibody (1 : 1000) for 1 h at 23°C. Protein detection was performed as described above.

## 3. Results

### 3.1. *Caecm33* Mutant Formed Less Biofilms

**Figure 1:**
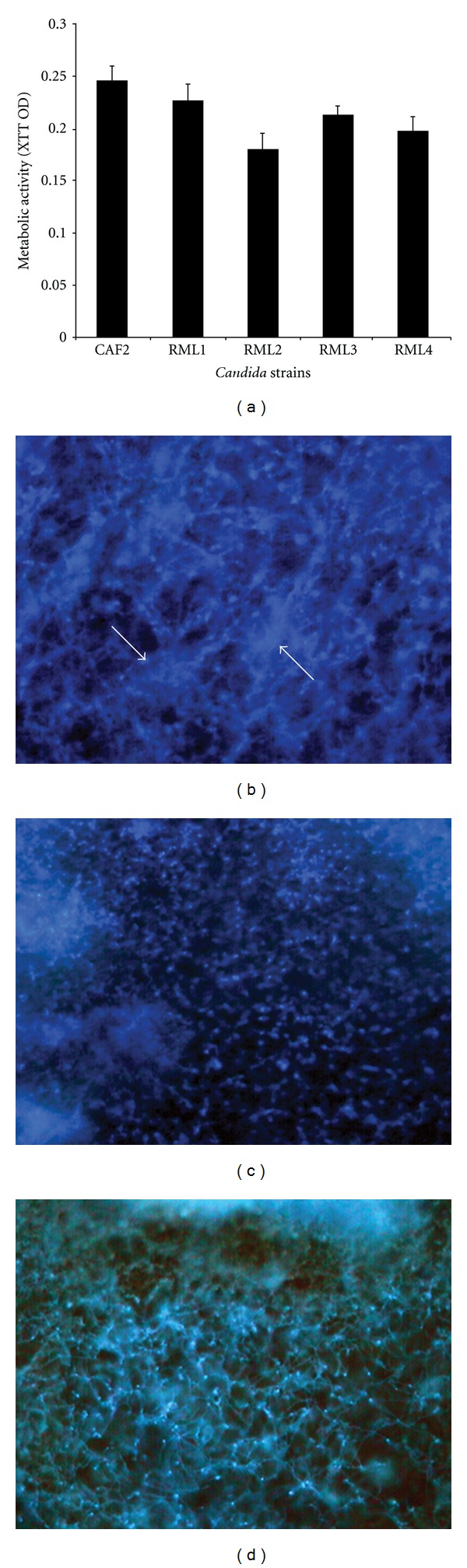
Biofilm-forming ability of isogenic wild-type, *Caecm33* mutant, and revertant strains. (a) Metabolic activity of mature biofilms formed by the isogenic strains. (b–d) Fluorescence micrographs showing differences in morphology of biofilms formed by (b) wild-type, (c) *ECM33* mutant, or (d) revertant strain. RML1-heterozygous *Caecm33/CaECM33* mutant; RML2-homozygous *Caecm33* mutant; RML3-heterozygous *Caecm33/CaECM33* revertant; RML4-homozygous *ECM33/ECM33* revertant.

 Our findings show that homozygous *Caecm33* mutant tended to form less biofilms than did the isogenic wild-type strain (*P* = 0.052, Figure 1(a)). Fluorescence microscopy results also reveal that the biofilms formed by *Caecm33* mutant were less dense and contained patchy clusters of a diffuse extracellular matrix, compared to that observed in the isogenic wild-type and revertant strains (Figures 1(b)–1(d)).

### 3.2. Effect of the *Caecm33* Mutants on the EHOM Structure

**Figure 2:**
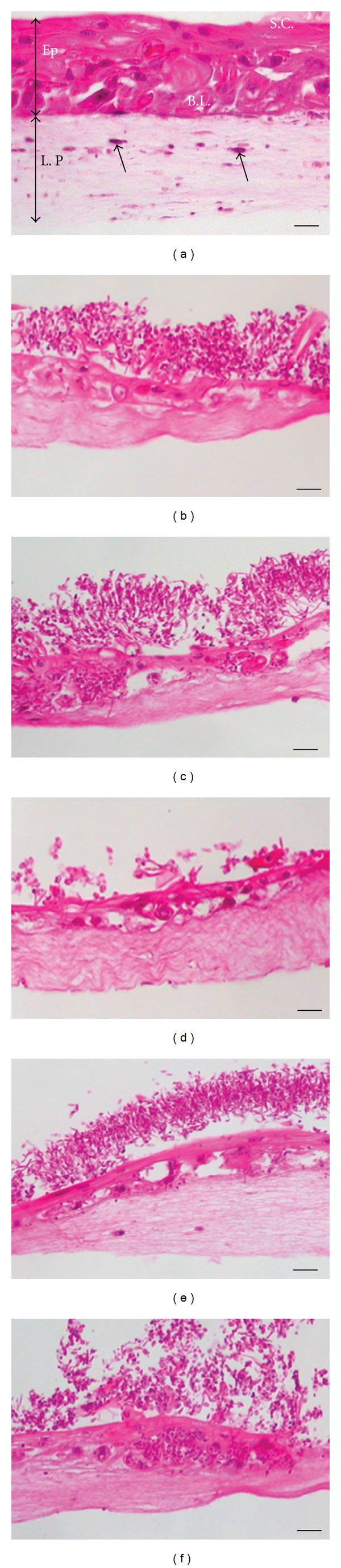
Interaction of *ECM33* mutants with EHOM and biofilm formation. Histological features of the EHOM following 24 h of exposure to each *C. albicans* strain. (a) Noninfected EHOM tissue; (b) infected with CAF2 strain; (c) RML1; (d) RML2; (e) RML3; (f) RML4 *C. albicans*. The noninfected EHOM (a) displays an epithelium (Ep) containing a stratum corneum layer (SC), a basal layer (BL), and a collagen-containing lamina propria (LP) populated with fibroblasts (arrows). Scale bars, 30 *μ*m. Representative photographs of three different experiments are shown (two EHOMs per experiment).

**Figure 3:**
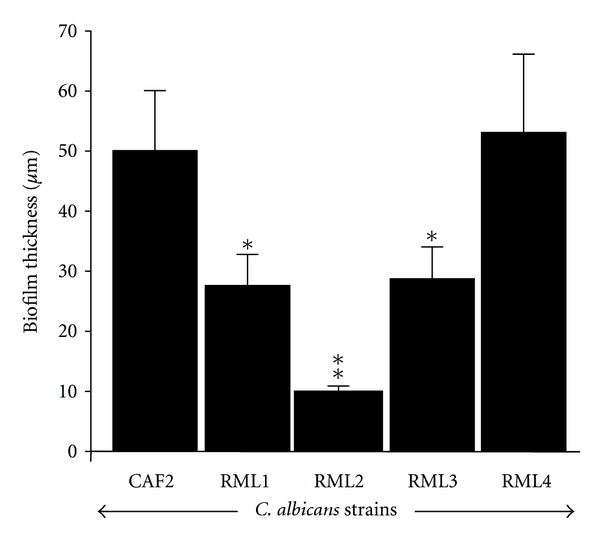
Effect of *ECM33* gene on the ability of *C. albicans* to form biofilm on EHOM. Following infection with each *Candida *strain and culture for 24 h, a quantitative assessment of the thickness of the biofilm formed on the EHOM tissues was performed. Means + SD (*n* = 4) were plotted. Differences were obtained by comparing the biofilms obtained with the CAF2 strain and the other *Candida* strains. *P < 0.05;  **P < 0.01.

The noninfected EHOM showed a well-organized epithelial structure characterized by different epithelial cell layers and a fibroblast-populated lamina propria (Figure 2(a)). Following infection with the CAF2 (Figure 2(b)) and RML4-homozygous revertant strains (Figure 2(f)), the EHOM displayed a thinner, more disorganized epithelium comprised of differentiated cells (i.e., large vacuolated cells containing a large nucleus). The mutation of one *Caecm33* allele partially prevented tissue damage (Figures 2(c) and 2(e)). The epithelium in the RML2-homozygous double *Caecm33* mutant-infected EHOM (Figure 2(d)) continued to display a stratified appearance. Close examination of the EHOM-infected tissues revealed the formation of biofilms containing both forms of *Candida* (yeast and hyphae) on the outer layer of the epithelium (Figure 2). Biofilm analysis showed that the CAF2 and RML4-homozygous revertant *Candida* strains formed significant biofilms on the EHOM layers, resulting in disorganized tissue layers, whereas the RML1-heterozygous *Caecm33* mutant, the RML2-homozygous double *Caecm33* mutant, and the RML3-heterozygous revertant strains all formed less biofilms on the EHOM epithelium (Figure 2), thus causing less damage. Quantitative analysis revealed that the biofilms formed by the *Caecm33* mutants were thinner than those formed by the CAF2 and RML4-homozygous revertant strains (Figure 3). Of interest is that the thinnest biofilms were obtained with the RML2*-*homozygous double *Caecm33* mutant strain. These studies show that a disruption of the *ECM33* gene resulted in a diminished ability of *C. albicans* to form biofilms (Figure 3) and to damage the host mucosal tissue.

### 3.3. Toxicity of the *Caecm33* Mutants on the EHOM Tissue

**Figure 4:**
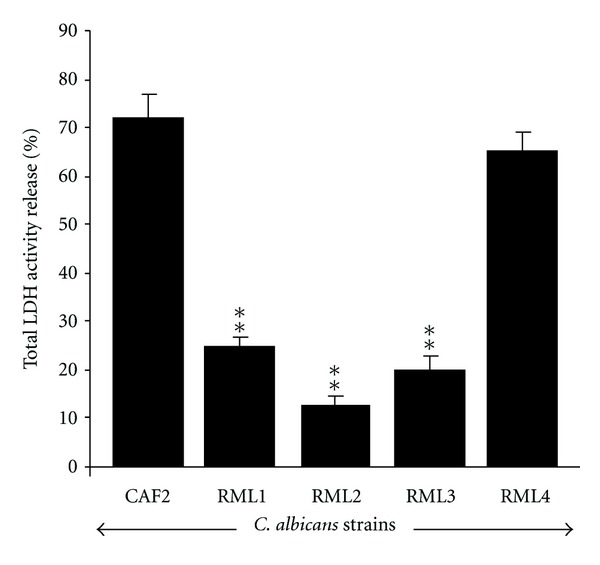
LDH release following EHOM infection with *C. albicans* strains. Culture media were collected from the noninfected and infected EHOMs. Supernatants were used to assess LDH release by means of specific kits. Data are means + SD, *n* = 5.  **P < 0.01 compared to the control (CAF2-infected EHOM).

As *Caecm33* mutants led to less tissue damage and reduced biofilm formation on the EHOM, we investigated the effect of each *Candida* strain on the release of LDH by the EHOM cells. It was observed (Figure 4) that infection of the EHOM with the CAF2 strain and the RML4-homozygous revertant strain resulted in high levels of LDH in the medium, which is in agreement with our observations of the damaged epithelium (Figure 2) obtained with the CAF2 strain. It should be noted that each* Caecm33 *mutant negatively affected LDH release by the EHOM compared to that observed with the CAF2 strain (Figure 4).

### 3.4. Apoptotic Gene Expression and Protein Production by the EHOM following Infection with *Caecm33* Mutant *C. albicans*

**Figure 5:**
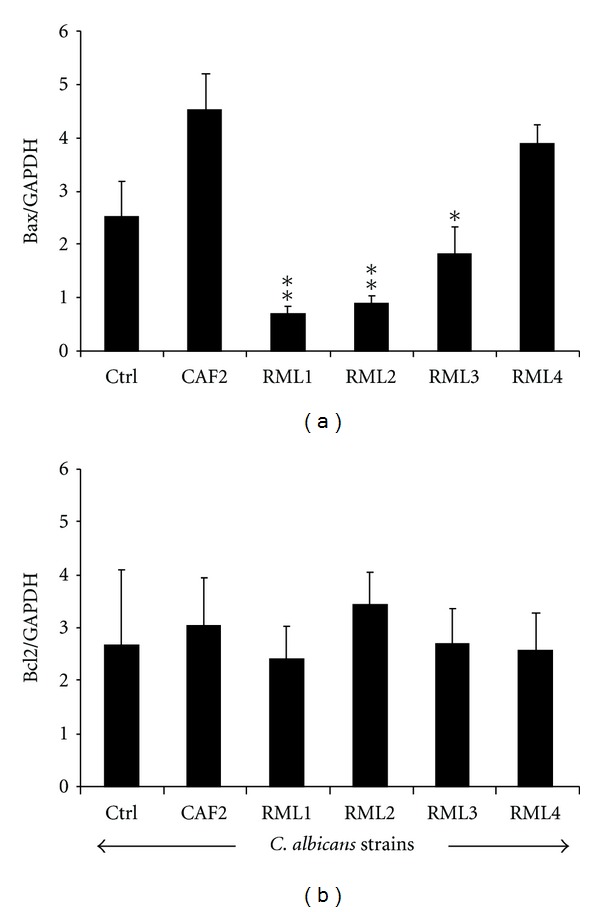
Bax and Bcl2 expression following EHOM infection with *C. albicans* strains. Total RNAs were extracted from the infected EHOMs and analyzed by RT-PCR for Bax and Bcl2 gene expression. Data are means + SD, *n* = 5.  *P < 0.05;  **P < 0.01 significance between the gene expression levels in the CAF2-infected and *Caecm33* mutant-infected EHOMs.

**Figure 6:**
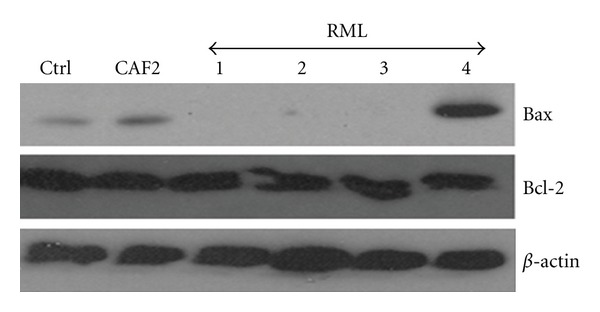
Bax and Bcl2 protein analyses following EHOM infection with *C. albicans* strains. Total proteins were extracted from the infected EHOMs and analyzed by western blotting for Bax and Bcl2 production. Gels are representative (*n* = 5).

The CAF2 and RML4-homozygous revertant strains significantly increased the Bax/Bcl2 gene expression ratio compared to that recorded by the noninfected EHOM. This was due to increased Bax gene expression which is an indicator of cell apoptosis. Following EHOM infection with the *Caecm33* mutants, Bax gene expression significantly (*P* < 0.01) decreased compared to that observed with the CAF2-infected tissue (Figure 5(a)). Furthermore, *Caecm33 *double mutant recorded the lowest level of Bax gene expression. RT-PCR analyses of Bcl2 gene showed that its expression remained unchanged following tissue infection with all of the *Candida* strains under study (Figure 5(b)). These findings are supported by Bax and Bcl2 protein analyses using western blotting.  Figure 6 shows that Bcl2 was unchanged; however, Bax protein was highly present in the EHOM infected with the CAF2 and RML4-homozygous strains compared to the noninfected and *Caecm33* mutant-infected specimens. Overall data indicate that apoptotic genes and proteins' levels of expression increased when the tissues were infected with CAF2. Infection with the mutant strains nevertheless showed a reduction in apoptotic gene and protein expression, which may prevent mammalian cell death/apoptosis.

### 3.5. The *C. albicans* Strains Modulated Laminin 5 and Type IV Collagen Synthesis

**Figure 7:**
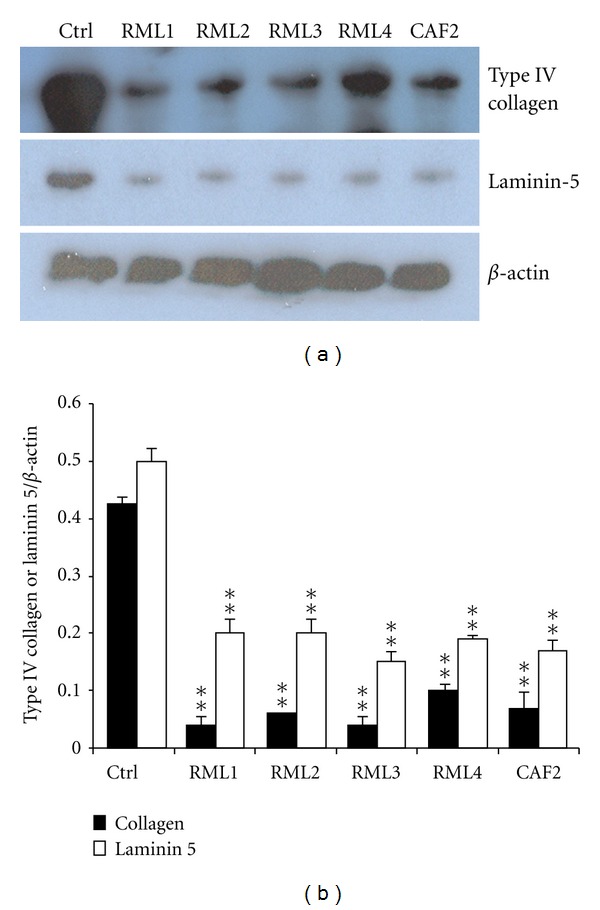
*C. albicans* downregulated basement membrane protein production. Total proteins were extracted from the infected EHOMs and analyzed by western blotting for the presence of laminin 5 and type IV collagen. (a) Representative gel of three separate experiments. Unstimulated tissue was used as a control (Ctrl). Protein production modulation was determined by band scanning using public domain NIH Image software. (b) Histograms are the means + SD (*n* = 4). Statistical differences were found by comparing the values obtained from the infected and noninfected tissues. **P < 0.01.

Western blot results show elevated basal levels of laminin 5 and type IV collagen proteins in the noninfected EHOM (Figure 7(a)), whereas both proteins were significantly (*P* < 0.05) down regulated in the infected tissues. It is important to note that all of the tested *Candida* strains significantly decreased laminin 5 and type IV collagen levels in the infected EHOM (Figure 7(b)) compared to the noninfected specimens. Thus *ECM33* gene mutation did not prevent the laminin 5 and type IV collagen decreases.

## 4. Discussion

*C. albicans* adhesion to various surfaces, including mucosal tissue and catheter disks, is the first step in biofilm formation and host infection [32, 33]. Our study demonstrates that the *Candida* adhesion to and biofilm formation on catheter discs were modulated by *ECM33* gene, and that the disruption of this gene led to decreased biofilm formation, which supports previously reported data with medical devices [3, 34]. Thus adhesion and biofilm formation on a catheter that is in contact with host tissue may contribute to tissue disorganization and facilitate yeast penetration into the deep tissue and subsequent systemic invasion [35].

*C. albicans* adhesion to mammalian cells and tissue is under the control of various genes, such as *ECM33*. Using an EHOM model, we demonstrated that *Caecm33* mutant was unable to damage tissue structure, compared to the CAF2 strains. This inability to cause tissue damage may be linked to a defect in the ability of *Caecm33* mutant to form hyphae. Indeed, it was reported that *Caecm33* mutants displayed filamentation defects in both liquid and solid media [17]. The inability of *ECM33* mutant to damage the EHOM tissue may also be due to cell wall architectural changes that hamper interactions with host cell/tissue. *Caecm33* mutants have in fact been shown to exhibit significant cell changes, including an abnormally electron-dense outer mannoprotein layer [17]. This architectural modification likely contributed to the reduced adherence level, decreased biofilm formation, and less extensive tissue damage observed in this study.

*Caecm33* mutants were also less capable of promoting cell death/apoptosis by reducing LDH release and Bax gene expression. These findings support the reduced tissue disorganization when placed in contact with the *Caecm33* mutants. Recent studies suggest that the decreased host cell damage caused by *Caecm33* mutants is likely due in part to the reduced endocytosis of these strains [8]. *Caecm33* mutant strains may also secrete less lytic enzymes, such as secreted aspartyl proteases and phospholipases [8], thus contributing to the host tissue cell damage defect of these mutants. Further studies are necessary to determine the mechanisms underlying the decreased virulence of the *Caecm33* mutant strains.

EHOM disorganization following contact with the *ECM33*-positive gene may also modulate some physiological mechanisms such as BM protein production. Indeed, following contact with the CAF2 and *Caecm33* mutant strains, both laminin 5 and type IV collagen levels decreased significantly. By decreasing BM protein production, *Candida* may thus deregulate epithelial and connective tissue structural interactions, thereby reducing their capacity to prevent its invasion [36]. It has been shown that *Candida* invasion requires adhesion to BM proteins as well as their degradation [21]. Although *Candida* is capable of degrading these proteins, this does not necessarily mean that *Caecm33* mutant will, in turn, downregulate laminin 5 and type IV collagen protein production. The deletion of single or double *Caecm33* alleles led to the same effect as that produced by the CAF2 strains on BM protein expression. This suggests that *ECM33* mutation does not reduce the ability of *Candida* to decrease BM protein synthesis by gingival cells. Further research will be conducted to shed light on this possible mechanism.

In conclusion, we used an engineered human oral mucosa model to demonstrate that *Caecm33* mutant was not able to damage tissue structure and promote cell apoptosis. We also demonstrated that biofilm formation was reduced by *Caecm33* mutants compared to parental strains. Finally, we showed that parental and *Caecm33* mutant strains downregulated laminin 5 and type IV collagen production. Overall data thus suggest that *ECM33* gene plays an active role in *Candida-*host interactions and is responsible for tissue damage that ultimately leads to candidiasis.

## Figures and Tables

**Table 1:** *Candida* strains used in this study.

Strain	Relevant characteristics	Reference
CAF2-parental strain	*URA3*/Δ*ura3*::*imm43 *	[16]
RML1-heterozygous *ecm33* mutant	*CaECM33/*Δ*Caecm33:: hisG-CaURA3-hisG *	[17]
RML2-homozygous double *ecm33* mutant	Δ*Caecm33*Δ*:: hisG/Caecm33*Δ*:: hisG-CaURA3-hisG *	[17]
RML3-heterozygous revertant	*Caecm33*Δ*:: hisG/CaECM33-clz-URA3-clz-hisG *	[17]
RML4-homozygous revertant	*CaECM33-clz-hisG/CaECM33-clz-URA3-clz-hisG*	[17]

**Table 2:** Description of oligonucleotide primer pairs used in PCR reactions.

Gene	Primer sequence (5′ to 3′)	Amp size (bp)
Bax	Sens 5′-TGG CAG ACA TGT TTT CTG AC-3′	204
	Antisens 5′-TCA CCC AAC CAC CCT GGT CTT-3′	
Bcl2	Sens 5′-TTT GAG TTC GGT GGG GTC AT-3′	274
	Antisens 5′-TGA CTT CAC TTG TGG CCC AG-3′	
GAPDH	Sens 5′-ATGCAACGGATTTGGTCGTAT-3′	221
	Antisens 5′-TCTCCTCCTGGAAGATGGTG-3′	
